# Emerging signs of Alzheimer‐like tau hyperphosphorylation and neuroinflammation in the brain post recovery from COVID‐19

**DOI:** 10.1111/acel.14352

**Published:** 2024-09-29

**Authors:** Xuetao Qi, Shulu Yuan, Jiuyang Ding, Weiqi Sun, Yajiao Shi, Yuanwei Xing, Zilong Liu, Yun Yao, Su Fu, Baofei Sun, Xiaolan Qi, Bing Xia, Fengyu Liu, Ming Yi, Jian Mao, You Wan, Jie Zheng

**Affiliations:** ^1^ Neuroscience Research Institute and Department of Neurobiology, School of Basic Medical Sciences Peking University Beijing China; ^2^ Key Laboratory for Neuroscience, Ministry of Education/National Health Commission Peking University Beijing China; ^3^ Key Laboratory of Human Brain Bank for Functions and Diseases of Department of Education of Guizhou Province Guizhou Medical University Guiyang China; ^4^ Key Laboratory of Endemic and Ethnic Diseases, Ministry of Education Guizhou Medical University Guiyang China; ^5^ School of Forensic Medicine Guizhou Medical University Guiyang China; ^6^ Beijing Life Science Academy Beijing China

**Keywords:** Alzheimer's disease, COVID‐19, glia cell, inflammation, tau

## Abstract

Coronavirus disease 2019 (COVID‐19) has been suggested to increase the risk of memory decline and Alzheimer's disease (AD), the main cause of dementia in the elderly. However, direct evidence about whether COVID‐19 induces AD‐like neuropathological changes in the brain, especially post recovery from acute infection, is still lacking. Here, using postmortem human brain samples, we found abnormal accumulation of hyperphosphorylated tau protein in the hippocampus and medial entorhinal cortex within 4–13 months post clinically recovery from acute COVID‐19, together with prolonged activation of glia cells and increases in inflammatory factors, even though no SARS‐COV‐2 invasion was detected in these regions. By contrast, COVID‐19 did not change beta‐amyloid deposition and hippocampal neuron number, and had limited effects on AD‐related pathological phenotypes in olfactory circuits including olfactory bulb, anterior olfactory nucleus, olfactory tubercle, piriform cortex and lateral entorhinal cortex. These results provide neuropathological evidences linking COVID‐19 with prognostic increase of risk for AD.

## Graphical abstract

Abnormal accumulation of hyperphosphorylated tau protein, together with prolonged activation of glia cells and increases in inflammatory factors in the hippocampus and medial entorhinal cortex post clinically recovery from acute COVID‐19.

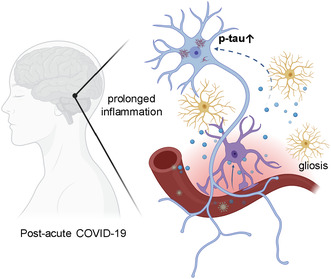

**Abbreviations**
AD: Alzheimer's disease
AON: anterior olfactory nucleus
Aβ: amyloid‐beta
COVID‐19: Coronavirus disease 2019
ddPCR: droplet digital PCR
GFAP: glial Fibrillary acidic protein
HMGB1: high mobility group box 1
IL‐10: interleukin‐10
IL‐18: interleukin‐18
IL‐1β: interleukin‐1β
IL‐6: interleukin‐6
LEC: lateral entorhinal cortex
MEC: medial entorhinal cortex
NAAT: nucleic acid amplification test
OB: olfactory bulb
OT: olfactory tubercle
P1~P4: pattern 1~4
PAI1: plasminogen activator inhibitor‐1
Pir: piriform cortex
pTau: phosphorylated tau
TNF‐α: tumor necrosis factor alpha

Cognitive decline including memory problems, mental cloudiness and difficulty in concentrating (collectively known as “brain fog”) has been widely recognized as parts of neuropathological sequelae of coronavirus disease 2019 (COVID‐19) in recent years (Thaweethai et al., 2023), and captures increasing attention on whether and how the history of Severe acute respiratory syndrome coronavirus 2 (SARS‐CoV‐2) infection would increase the risk of Alzheimer's disease (AD) in later life, especially for the elderly (Bonhenry et al., 2024; Olivera et al., 2023). However, direct evidence on the appearance of AD‐related neuropathological features, like abnormal accumulation of hyperphosphorylated tau protein and amyloid‐beta (Aβ) as well as glia dysfunction and neuroinflammation in the brain (Long & Holtzman, 2019), in human brain post recovery from acute COVID‐19 is still in lack.

Here, we collected postmortem human brain samples from individuals died with SARS‐COV‐2 non‐infected (group “N”, *n* = 6), during acute COVID‐19 (group “C”, n = 6), and within 4–13 months post‐clinically recovered from COVID‐19 (group “R”, *n* = 7). Only individuals who had no cognitive impairment reported before SARS‐COV‐2 infection were included, and the infection and recovery of SARS‐COV‐2 was determined or ruled out by nucleic acid amplification test (NAAT). Individual information was summarized in Table S1.

We first examined the level of phosphorylated tau (pTau) in the hippocampus and medial entorhinal cortex (MEC), since these regions play pivotal roles in the development of AD, and have been well‐recognized to be vulnerable for pTau accumulation and neurodegeneration (Braak et al., 2006). Tau species ranging from 35 to 100 kD and phosphorylated at multiple AD‐related epitopes were measured. Interestingly, we found that both pTau and total tau showed nonsignificant change in patients died during acute COVID‐19 compared with individuals without SARS‐COV‐2 infection, but pTau was unexpectedly upregulated in the group clinically recovered from COVID‐19, especially at AD‐related epitopes like Thr181, Thr217 and AT8 (Figure 1a–c, Figure S1).

**FIGURE 1:**
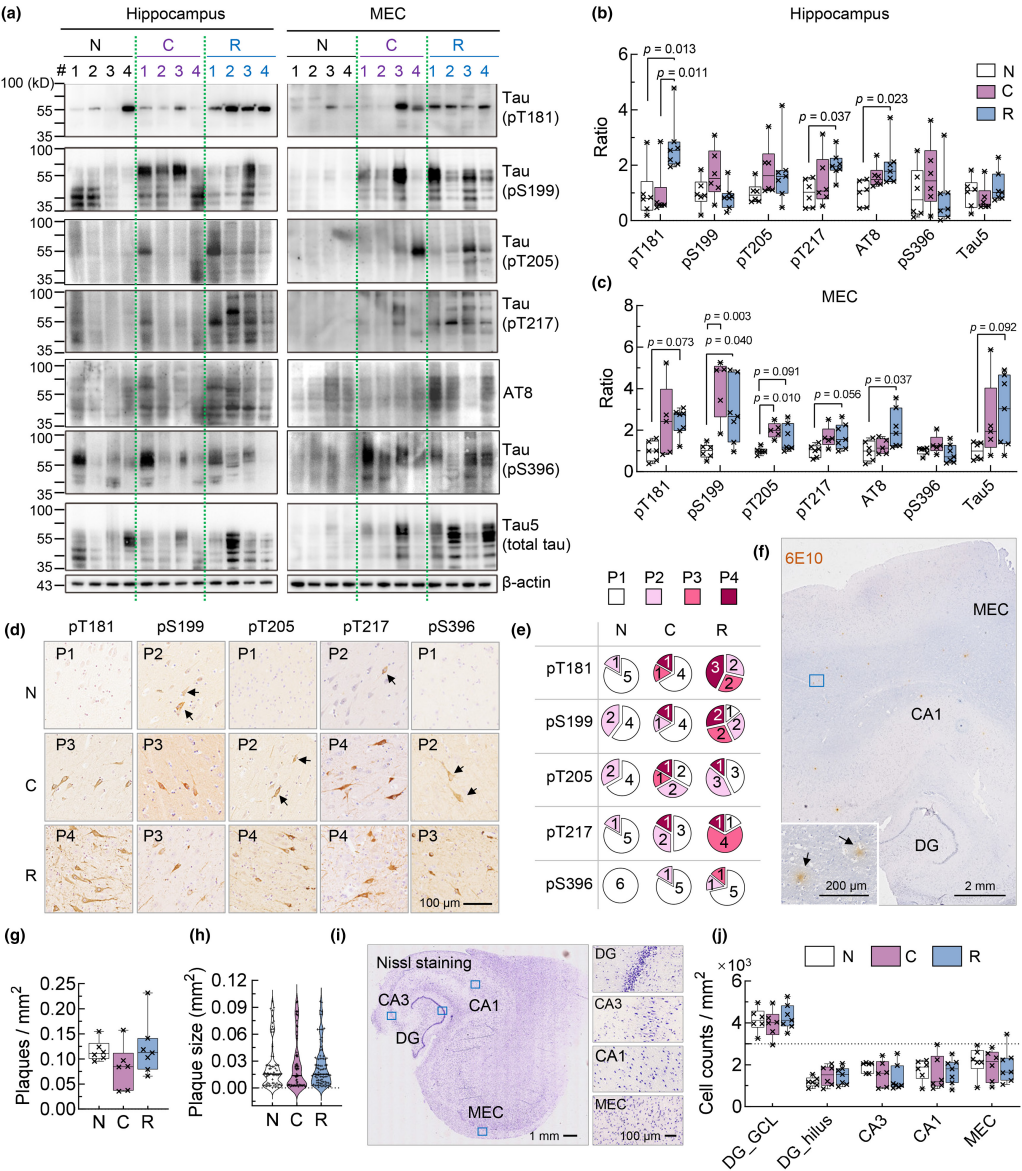
Upregulation of pTau in the hippocampus and MEC post COVID‐19. (a–c) pTau at specific epitopes like Thr181, Thr217 and AT8 were upregulated in the hippocampus and MEC post‐acute COVID‐19. Grouping: N, non‐infected; C, acute COVID‐19; R, recovery from COVID‐19. *N* = 6–7 in each group, one‐way ANOVA followed by Tukey's multiple comparisons tests. Data were normalized by the mean value of group N for each pTau epitope. Extended blots were shown in Figure S1. (d, e) Measurement of pTau distribution pattern in the hippocampus and MEC indicated increase of pTau aggregation post‐acute COVID‐19. Representative images showed pattern 1–4 (P1–P4) of pTau accumulation for each epitope and in each group (d). Numbers in pie charts indicated the counts of patients for each pattern (e). *N* = 6–7 in each group. (f–h) The overall density and averaged size of 6E10‐stained Aβ plaques did not change in acute and post‐acute COVID‐19. *N* = 6–7 in each group, one‐way ANOVA followed by Tukey's multiple comparisons tests. (i, j) Neuronal density in each hippocampal subregion did not change in acute and post‐acute COVID‐19. *N* = 6–7 individuals in each group, one‐way ANOVA followed by Tukey's multiple comparisons tests.

Meanwhile, we also evaluated the distribution pattern of pTau in the hippocampus and MEC, which were classified here into patterns 1–4 (P1–P4) based on the immunohistochemical staining intensity (supplementary methods—Data S1). The post‐acute COVID‐19 group generally exhibited higher degree of pTau aggregation compared with SARS‐COV‐2 non‐infected controls at Thr181 and Thr217 epitopes (Figure 1d,e).

We next examined two other AD‐related pathologies including Aβ deposition and hippocampal neuron loss. By contrast to tau, no statistical change in the density and size of Aβ plaques was measured (Figure 1f–h), and no hippocampal neuron loss was found both during and post‐acute COVID‐19 (Figure 1i,j).

Besides, taken into consideration that COVID‐19 and early‐stage AD shared symptom of smell loss (Doty, 2022; Tsukahara et al., 2023), a newly‐identified biomarker of cognitive impairment (Murphy, 2019), we wonder whether acute or post‐acute COVID‐19 affects pTau and Aβ in the olfactory circuit consisting of olfactory bulb (OB), anterior olfactory nucleus (AON), olfactory tubercle (OT), piriform cortex (Pir) and lateral entorhinal cortex (LEC). By contrast to the hippocampus and MEC, no significant change in pTau and Aβ plaque was measured in all olfactory areas (Figure S2).

Subsequently, we sought to explore whether the elevation of pTau was associated with potential invasion of SARS‐COV‐2 into the brain, which was found to be capable of directly inducing tau hyperphosphorylation in cultured neuroblastoma cells and 3D human brain organoids (Di Primio et al., 2023; Ramani et al., 2020). However, we did not observe the existence of SARS‐COV‐2 in the brain utilizing immunohistochemical staining of nucleocapsid (N) and spike glycoprotein (S) protein (Figure S3). Consistently, we did not detect SARS‐COV‐2 ORFab1 and N protein mRNA by both real‐time quantitative reverse transcription PCR (RT‐qPCR) and droplet digital PCR (ddPCR) (Figures S4 and S5).

Given an important contribution of abnormal glia activation and neuroinflammation to the elevation of pTau in the development of AD (Leng & Edison, 2021), we measured whether COVID‐19 induced glia dysfunction in the hippocampus and MEC, and found increased soma volume of and less ramified processes of microglia during and post‐acute COVID‐19, two morphological signs indicating the activation microglia (Leng & Edison, 2021; Woodburn et al., 2021), though microglia did not change in number (Figure 2a–e). Similarly, the number of GFAP‐labeled astrocytes did not change, but both the soma volume and complexity of processes of astrocytes increased post recovery from COVID‐19 compared with acute COVID‐19 (Figure 2f–j), indicating that astrocytes were also activated during and post COVID‐19. Consistently, the activation of glia cells was also indicated by the upregulation in protein levels of Iba1, CD68, GFAP and S100β in groups of acute and post‐acute COVID‐19 (Figure S6).

**FIGURE 2:**
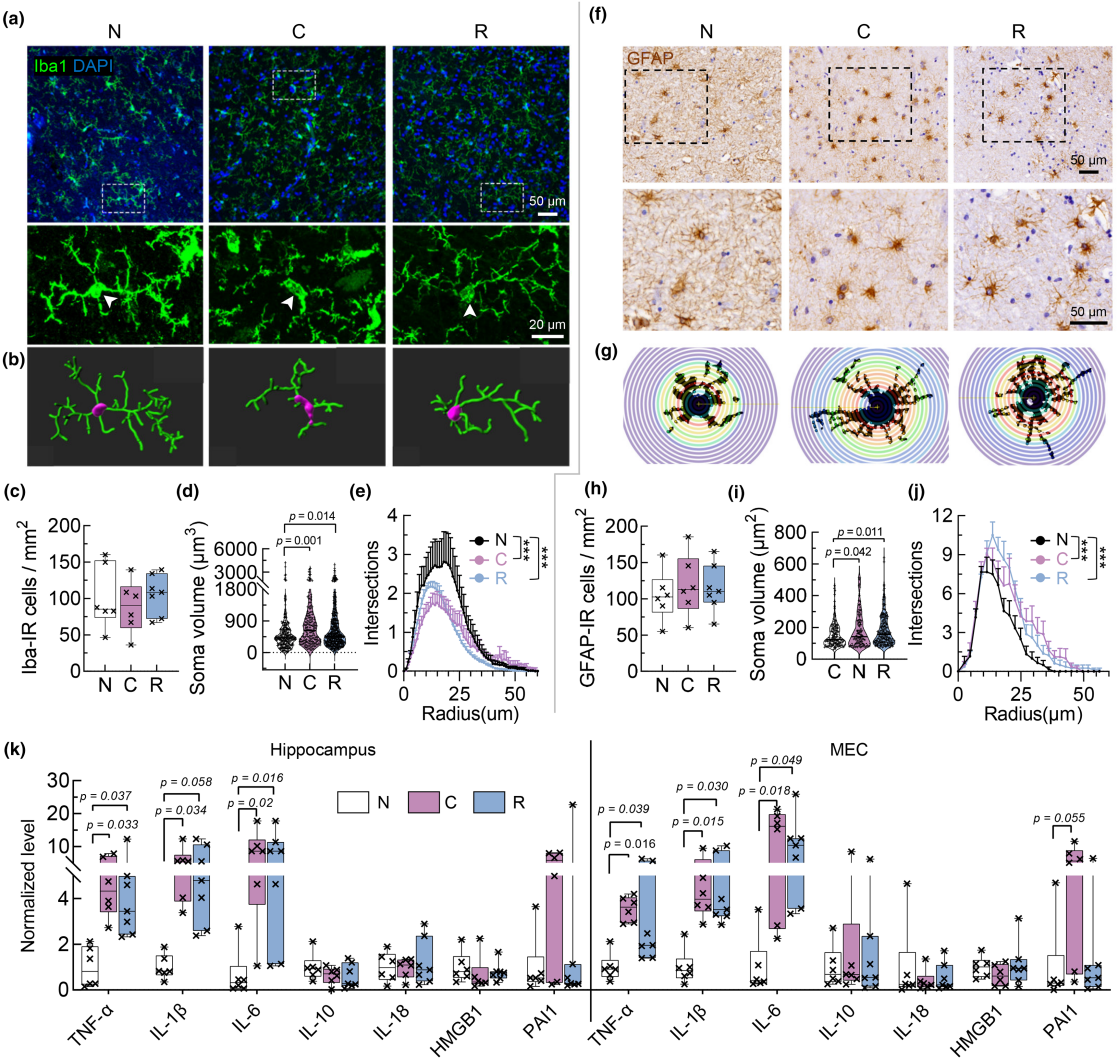
Prolonged upregulation of glia activation and inflammatory factors expression in the hippocampus post‐acute COVID‐19. (a, b) Representative immunofluorescent images (a) and 3D‐reconstruacted morphology (b) of Iba1‐stained microglia. (c–e) The number of microglia did not change (c), but soma volume significantly increased (d) and branches complexity decreased (e) in acute and post‐acute COVID‐19. *N* = 6–7 patients or 174–382 cells in each group, ***p < 0.001, one‐way (c, d) and repeated measures (e) ANOVA followed by Tukey's multiple comparisons tests. (f, g) Representative immunohistochemical images (f) and Sholl analysis diagram (g) of GFAP‐stained astrocytes. (h–j) The number of astrocytes did not change (h), but both soma volume (i) and processes complexity (j) significantly increased in acute and post‐acute COVID‐19. *N* = 6–7 patients or 108–194 cells in each group, ***p < 0.001, one‐way ANOVA followed by Tukey's multiple comparisons tests. (k) mRNA levels of inflammatory factors including TNF‐α, IL‐1β and IL‐6 were upregulated in acute and post‐acute COVID‐19. *N* = 6–7 patients in each group, one‐way ANOVA followed by Tukey's multiple comparisons tests.

Moreover, the expression levels of inflammatory factors including tumor necrosis factor alpha (TNF‐α), interleukin‐1β (IL‐1β) and interleukin‐6 (IL‐6) were all upregulated in acute and post‐acute COVID‐19, though other cytokines like interleukin‐10 (IL‐10) and interleukin‐18 (IL‐18), as well as neuroinflammation‐related proteins like high mobility group box 1 (HMGB1) and plasminogen activator inhibitor‐1 (PAI1) remained nearly unchanged (Figure 2k).

In line with our results, the risk of AD has been recently found to increase post‐acute COVID‐19 (Bonhenry et al., 2024). A follow‐up study found persist elevation of AD‐related plasma biomarkers including total‐tau, pTau181, inflammatory cytokines, NfL, neurogranin, etc. at 1 to 3 months after initial SARS‐CoV‐2 infection (Sun et al., 2021). Meanwhile, following with structural changes in brain during acute COVID‐19 (Douaud et al., 2022), persistent white matter changes in the brain of people recovered from COVID‐19 were also reported at 1‐year follow‐up (Huang et al., 2022). These findings suggest the existence of long‐term brain neuropathology after COVID‐19, which might be at least partly responsible for the post‐acute sequelae of cognition and memory impairment (Hampshire et al., 2024).

We sought to investigate in the present study preliminarily the potential cause of pTau elevation after COVID‐19. In discrepancy with several previous studies (Emmi et al., 2023; Gagliardi et al., 2021; Stein et al., 2022), we did not detect SARS‐COV‐2 particles in all brain regions measured in the present study. Instead, we revealed in individuals post‐acute COVID‐19 prolonged glia activation and neuroinflammation, two other important drivers of tau hyperphosphorylation in the development of AD (Chen & Yu, 2023; van der Kant et al., 2020). In fact, increased glia activation and inflammatory factors have been widely‐recognized both in the brain and plasma of COVID‐19 patients (Chen et al., 2020; Huang et al., 2020; Matschke et al., 2020; Zhang et al., 2023). The central neuroinflammatory infiltration might be a secondary consequence of the peripheral cytokine storm during acute SARS‐CoV‐2 infection due to the damage of brain–blood barrier (Amruta et al., 2022; Schwabenland et al., 2021). Excessive activation of glia cells and inflammation in the brain can exacerbate tau phosphorylation and aggregation by dysregulating tau kinases and phosphatases (Ising et al., 2019).

Nevertheless, our pilot study only included a small number of postmortem samples, it deserves further investigation to collect data from larger cohorts. Besides, it also remains to be elucidated whether and how long these AD‐associated pathological phenotypes persist following years to decades after recovery from acute COVID‐19, including the increased pTau level and neuroinflammation found here. The prognostic risk of AD might be better indicated through long‐term measurement of plasma or cerebrospinal fluid biomarkers (Ossenkoppele et al., 2022), brain imaging of early‐AD features (Jagust, 2018), and cognition‐testing scales (Woo et al., 2020).

In conclusion, we reported here emerging signs of AD‐like elevation of pTau and neuroinflammation during and post‐acute COVID‐19 in the hippocampus and MEC of postmortem human brain, which provided neuropathological evidences about increased risk of AD in long COVID‐19.

## AUTHOR CONTRIBUTIONS

*Conceptualization*: X. Q., J. D., J. Z; *Methodology*: X. Q., S. Y., J. D., Y. Y., S. F; *Investigation*: X. Q., S. Y., J. D., W. S., Y. S., Y. X., Z. L; *Data analysis*: X. Q., S. Y., J. Z; *Manuscript writing*: X. Q., J. Z; *Manuscript revision*: X. Q., B. S., X. Q., B. X., F. L., M. Y., J. M., Y. W., J.Z; *Funding acquisition*: J. M., Y. W., J. Z; *Resources*: J. D., Y. W., J. Z; *Supervision*: B. S., X. Q., B. X., Y. W., J. Z.

## CONFLICT OF INTEREST STATEMENT

All authors declare no conflicts interest.

## MATERIALS AND METHODS

Please see supplementary materials—Data S1.

## Supporting information


Data S1.

## Data Availability

All data were available by reasonable requirements to Dr. Jie Zheng (zhengjiie@hsc.pku.edu.cn).
