# Supplementary material for: Emerging signs of Alzheimer‐like tau hyperphosphorylation and neuroinflammation in the brain post recovery from COVID‐19
Source: Aging Cell. 2024 Sep 29;23(11):e14352. doi: 10.1111/acel.14352 (PMC11561645; doi:10.1111/acel.14352)
Supplement: Supplementary file 1 — Data S1. [file ACEL-23-e14352-s001.docx]

**Supplementary materials**

Supplementary figures S1~S6

Materials and methods

Supplementary table S1~S3


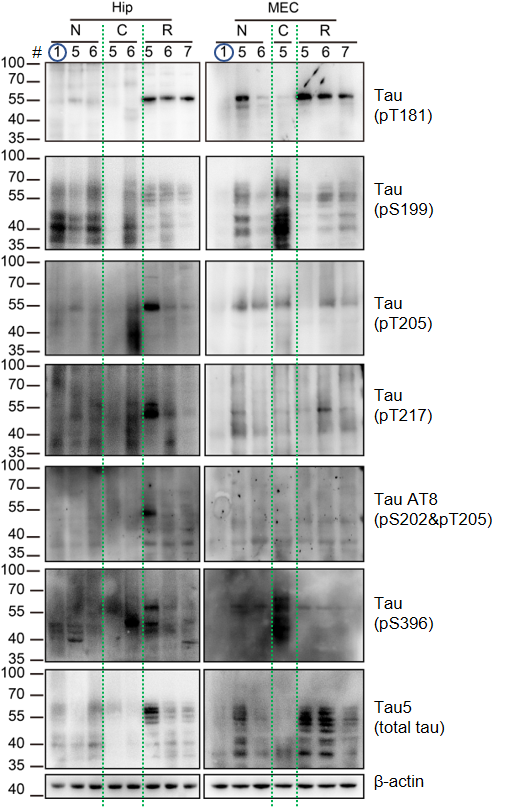


**Supplementary Fig. S1. Extended Western blotting results mearing tau in the hippocampus and medial entorhinal cortex.**

Sample for individual N#1 indicated in circle was re-loaded as control for quantification.


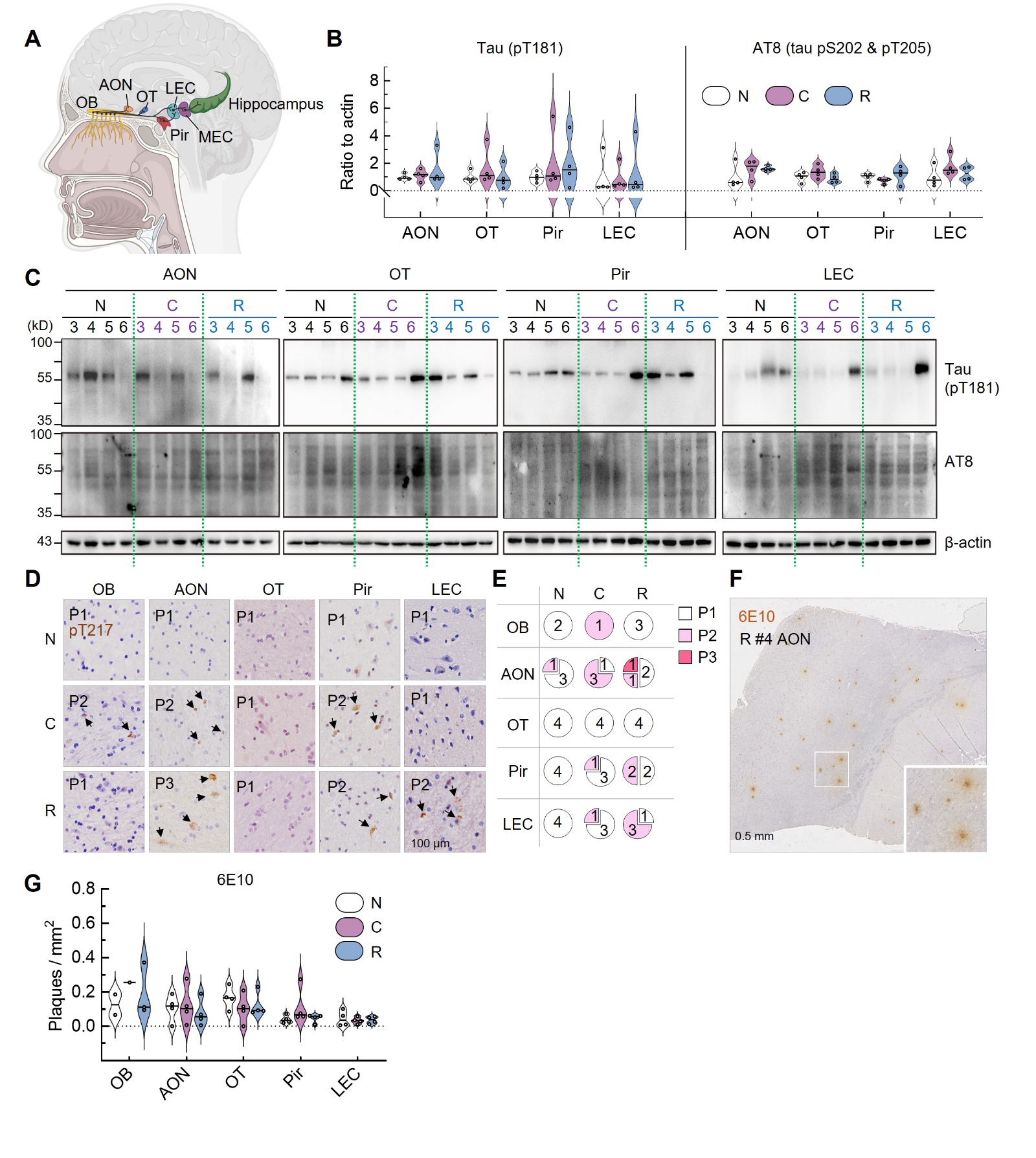


**Supplementary Fig. S2. Non-significant change in pTau and amyloid plaques in olfactory nuclei during and post-acute COVID-19**

**(A)** A cartoon illustrating olfactory circuits, hippocampus and MEC in human brain.

**(B, C)** pTau did not change in all olfactory nuclei during and post-acute COVID-19. N = 4 per group in each group, one-way ANOVA followed by Tukey’s multiple comparisons tests. Data were normalized by the mean value of group N for each pTau epitope.

**(D, E)** Distribution pattern of pTau in olfactory nuclei. Representative images showed the pattern 1~3 (P1~P3) of each epitope in each group (D). Numbers in pie charts indicated the counts of patients for each pattern (E). N = 1~3 per group for OB, and N = 4 per group for all other regions.

**(F, G)** 6E10-stained Aβ plaques did not change in acute and post-acute COVID-19. N = 1~3 per group for OB, and N = 4 per group for all other regions. one-way ANOVA followed by Tukey’s multiple comparisons tests.


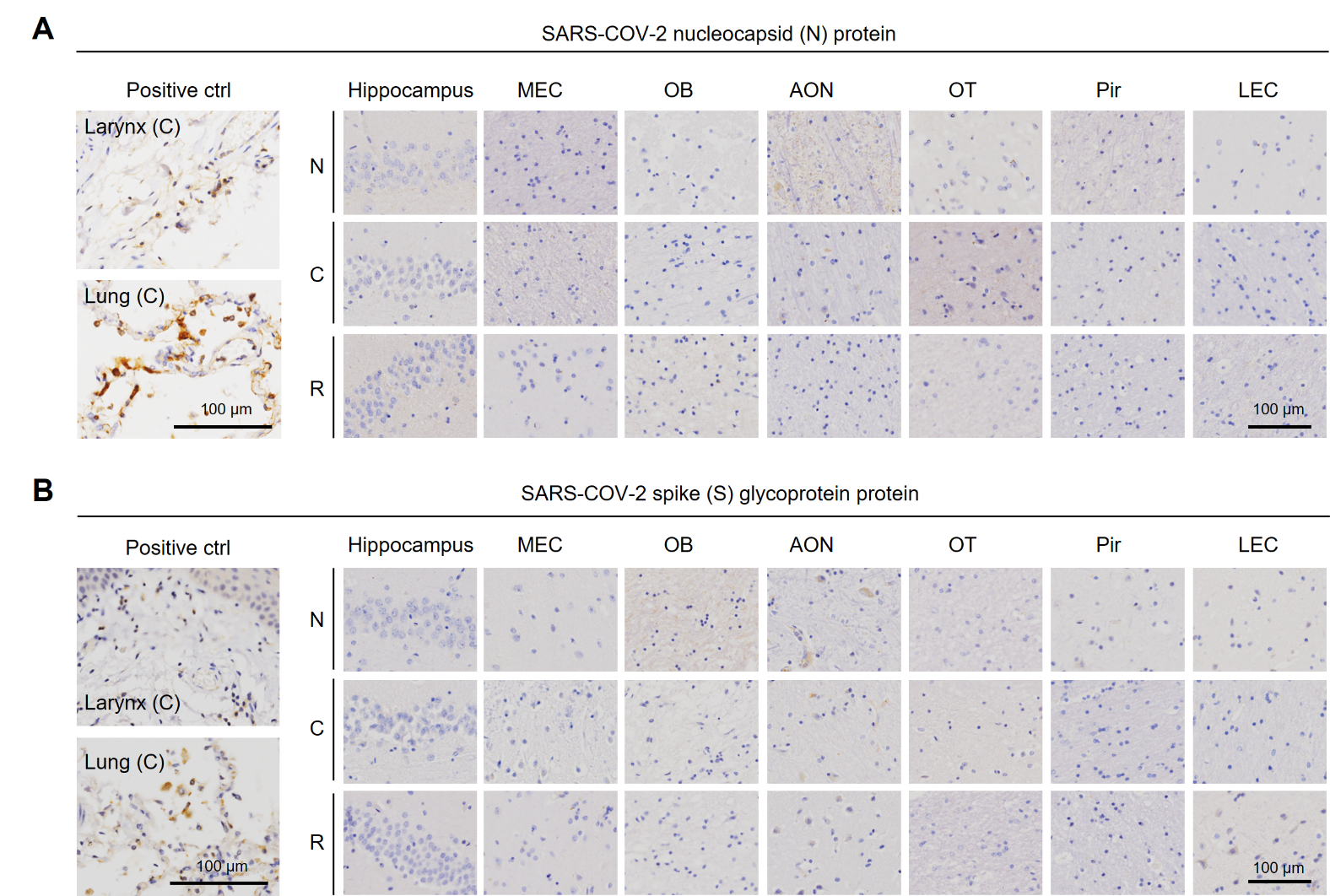


**Supplementary Fig. S3. No SARS-COV-2 protein in the brain is detected by immunostaining.**

No SARS-COV-2 N protein (A) and S protein (B) was detected by immunohistochemical staining in the brain. Larynx and lung tissue from patients died with serve COVID-19 were stained as positive control.


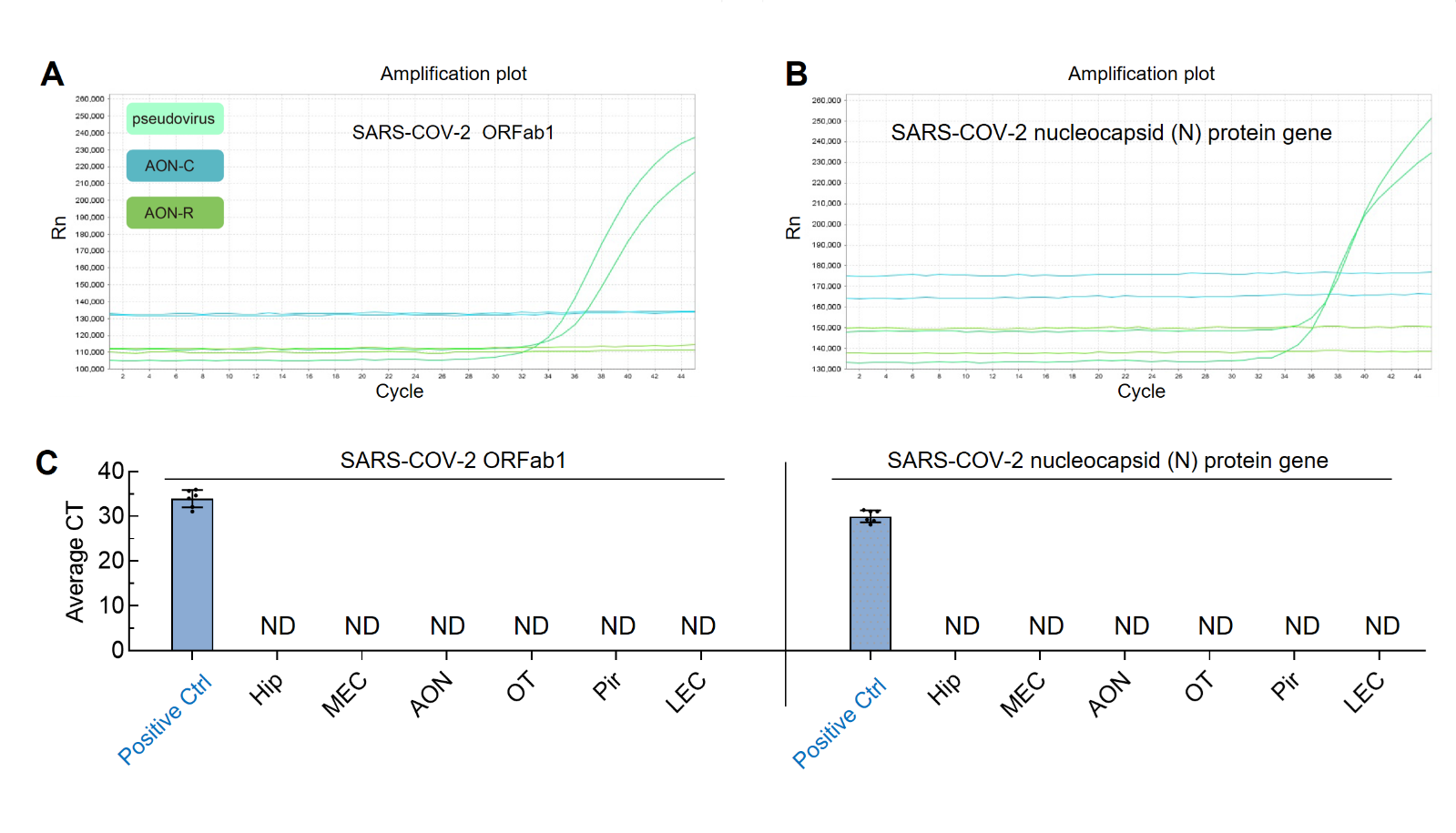


**Supplementary Fig. S4. No SARS-COV-2 mRNA in the brain is detected by RT-qPCR.**

**(A-B)** Representative RT-qPCR amplification plots of SARS-COV-2 ORF1ab1 (A) and N protein (B) mRNA for AON tissue and pseudovirus.

**(C)** No SARS-COV-2 ORF1ab1 and N protein mRNA was detected by RT-qPCR in the brain. ND: not detected. SARS-COV-2 pseudovirus was used as the positive control. N = 6 biological repeats or patients in each group.


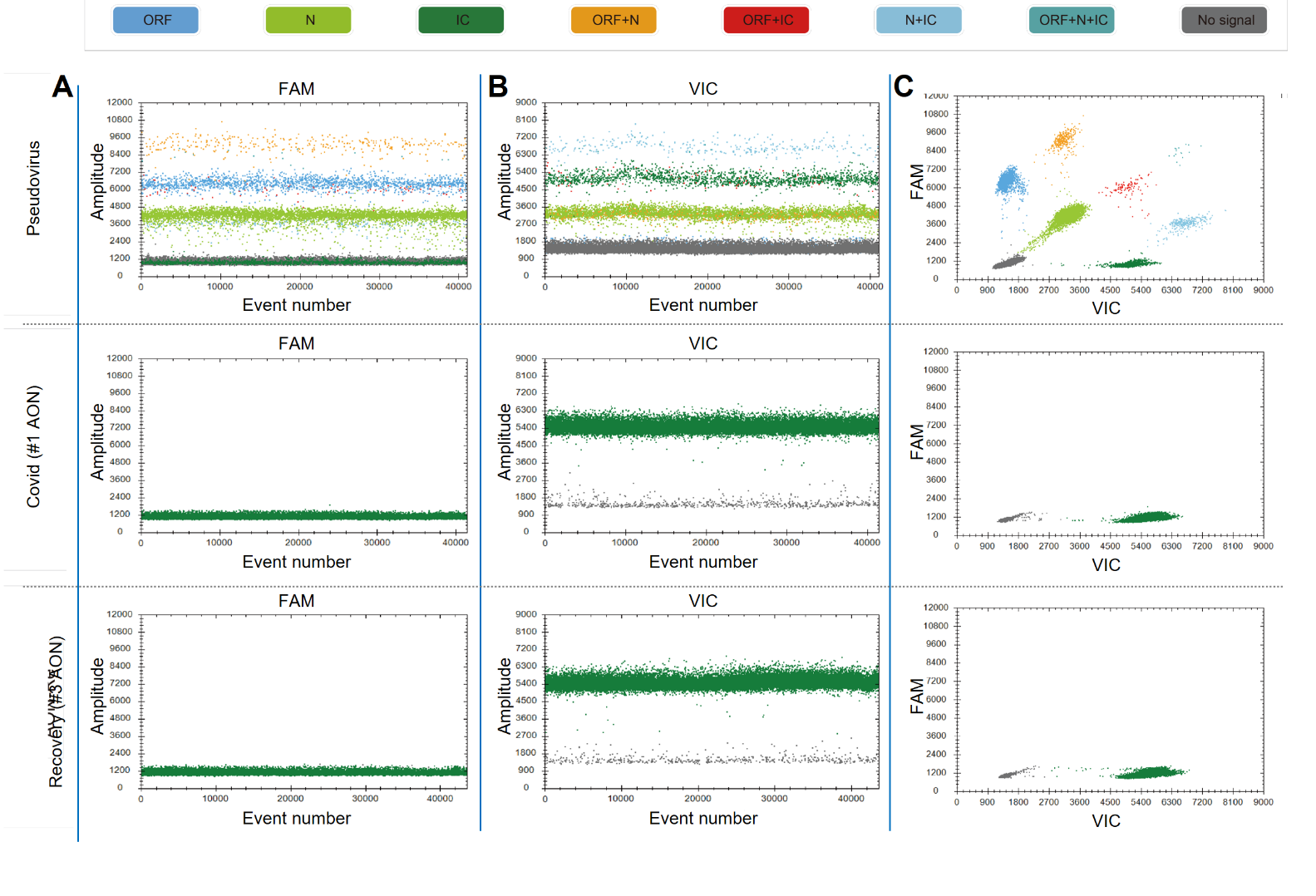
**Supplementary Fig. S5. No mRNA of SARS-COV-2 proteins in the AON is detected by ddPCR.**

**(A)** One-dimensional scatterplots of event number (droplets) vs. FAM fluorescence amplitude.

**(B)** One-dimensional scatterplots of event number vs. VIC fluorescence amplitude.

**(C)** Two-dimensional cluster plot in which FAM fluorescence is plotted against VIC fluorescence.

Different clusters are labeled and annotated by different colors. The appearance of certain cluster in two-dimensional plot indicates detectable expression of gene. IC, internal control. SARS-COV-2 pseudovirus was used as the positive control.


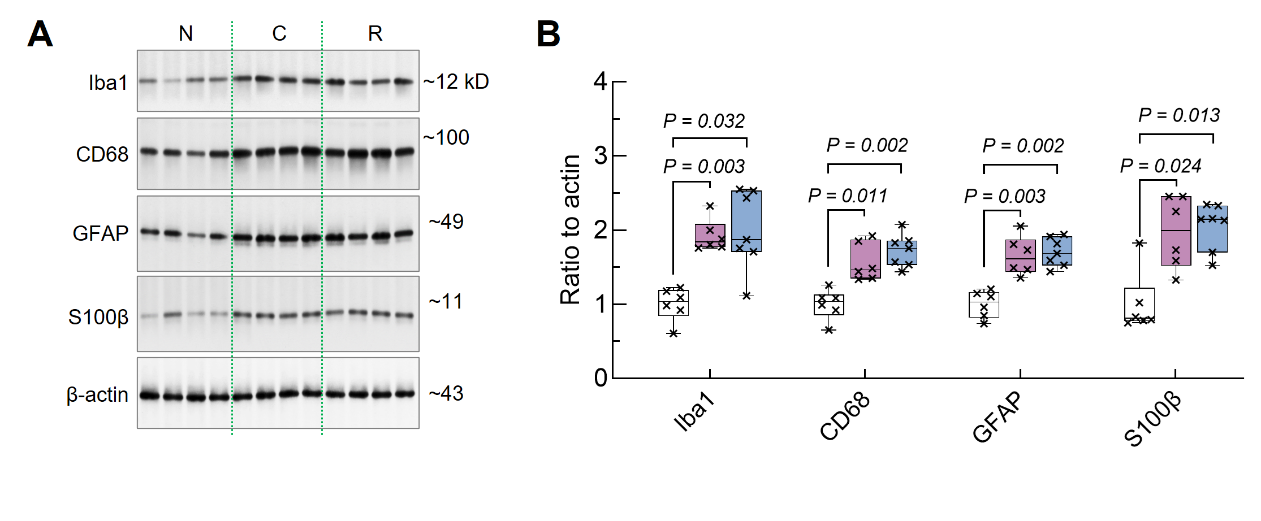


**Supplementary Fig. S6. Increased level of protein biomarkers for glia activation in the hippocampus of acute and post-acute COVID-19 patients.**

**(A)** Representative images of WB blots.

**(B)** Statistical results showing the upregulation of Iba1, CD68, GFAP and S100β, respectively, compared with non-infected controls. N = 6 per group in each group, one-way ANOVA followed by Tukey’s multiple comparisons tests. Data were normalized by actin and the mean ratio value of group N for each protein.

**Materials and methods**

**Human samples**

Human brain samples were collected from autopsies following consent of the legal next of kin. Individuals with no cognitive impairments report were included in the study. A total of 6 individuals of SARS-COV-2 **n**on-infected (group “N”), 6 patients died with diagnosed **C**OVID-19 (group “C”) within 2 weeks following SARS-COV-2 infection, and 7 individuals died for other reasons post-clinical **r**ecovery from COVID-19 (group “R”) with the recovery-death interval (RDI) of 4~13 months were included in this study. All individuals with diagnosed neurodegenerative diseases (like AD, Parkinson’s disease, etc.) or serve cognitive disorders (like non-AD types of dementia) before death were excluded from the sample collection. The potential infection of SARS-COV-2 for each person included was determined or ruled out by nucleic acid amplification test (NAAT). Brain tissues from hippocampus, medial entorhinal cortex, olfactory bulb, anterior olfactory nucleus, olfactory tubercle, piriform cortex and lateral entorhinal cortex from each individual were obtained at the time of autopsy for this study. Larynx and lung tissues from other two serve COVID-19 patients were obtained and used as the positive control of SARS-COV-2 proteins staining. Detailed information was summarized in supplementary Table S1.

For each brain region, tissues were dissected at comparable coronal levels among different participants. A part of fresh sample was rapidly frozen using liquid nitrogen and stored under -80 ℃, the remaining part was preserved for histopathological and biochemical staining, which was post-fixed at 4 ℃ in 4% neutral-buffered formalin for 14 days, then transferred to 70% ethanol and impregnated with paraffin. All experiments were approved by the Ethics Committee of Guizhou Medical University and Ethics Committee of Peking University Health Science Center.

**Western blotting (WB)**

Equal weight of tissue was extracted from hippocampal dentate gyrus, CA3 and CA1 respectively using a #9 puncture needle in cryostat microtome, and then mixed for homogenization using RIPA buffer (P0013B, Beyotime) containing 50mM Tris-HCl, 100mM NaCl, 1% Triton X-100, 5mM EDTA, and 1:100 PMSF and phosphatase inhibitor cocktails (P1046, Beyotime), and then centrifuged at 12000 g for 5 minutes. Protein concentrations in supernatants were determined by BCA assay (23225, Thermo Scientific). Samples of an equivalent of 15 μg total protein was mixed with loading buffer (P0285, Boyetime), then separated in 8% SDS-PAGE gels and transferred onto nitrocellulose membrane (Millipore). The membranes were blocked with 5% BSA at room temperature for 1 h, incubated with primary antibodies at 4°C for 12~36 h. After being washed in tris-buffered saline and 1% tween-20, the membranes were again incubated with horseradish peroxidase-conjugated secondary antibodies at room temperature for 1 h. Protein bands were detected using ECL luminol reagent (P0018S, Beyotime). Images were taken by a chemiluminescence image analysis system (Tanon5200, Tanon), and eventually quantified using Fiji software^[1]^. Antibodies used in the present study were summarized in supplementary Table S2.

**Immunohistochemical staining**

Formalin-fixed tissue was dehydrated and impregnated with paraffin via a full-automatic tissue processor according to the guideline of manufacturer (ASP300S, Lecia), and 5-µm sections was obtained using a microtome (HistoCore AUTOCUT, Lecia). Sections were deparaffinized in a series of ethanol, rehydrated in 0.01 M PBS for 10 minutes. Subsequently, sections were subjected to sodium citrate buffer (10 mM, pH 6.0) for 20 min at 95 °C for heat-induced epitope retrieval. Slides were then rinsed briefly in deionized water, incubated in 0.5% H_2_O_2_ for 10 min to inactivate endogenous peroxidase, washed with PBS and then blocked in 5% BSA for 30 min. Subsequently, sections were incubated in primary antibodies (diluted using blocking buffer) at 4℃ overnight, washed by PBS for 3×5 minutes, then incubated with peroxidase-conjugated secondary antibodies for 20 min at room temperature, followed by PBS washing for 3×5 minutes again. Immunoreactions were developed using a DAB-staining kit (DAB, PV-600D, ZSGB) according to manufacturer recommendations. Sections were rinsed with distilled water for about 10 seconds, counterstained with hematoxylin, dehydrated in a graded ethanol series, hyalinized in xylene series, and sealed with neutral balsam. All antibodies used in the present study were summarized in supplementary Table S2. Images were captured by digital slide scanner (NanoZoomer S60, HAMAMATSU) using a digital slide scanner (NanoZoomer S60, HAMAMATSU).

**Analysis of pTau aggregation**

The distribution pattern of pTau in the hippocampus and MEC were classified into patterns 1~4 (P1~P4): pattern 1 (P1) – no pTau-stained cells and neurites, P2 – pTau-stained cells sporadically distribute across the hippocampus and MEC (< 1 cell / mm^2^ on average) with moderate immunoreactivity, P3 – pTau-stained cells moderately cluster (1~10 cells / mm^2^ on average) with moderate immunoreactivity, P4 – pTau-stained cells widely distribute across the hippocampus and MEC ( > 10 cells / mm^2^ on average) with evident neurofibrillary tangles or neuritic threads-like morphology. Numbers of p-tau-positive cells across the whole hippocampal area were counted and average density were calculated.

**Nissl staining**

Slide-mounted sections were deparaffinized in ethanol, rehydrated in PBS in similar ways as for IHC staining, and then immersed in Nissl staining solution (C0117, Beyotime) for 7 min, rinsed with distilled water. Slides were then decolored and in turn in 50~100% ethanol, hyalinized in xylene for 2×10 minutes, and then mounted in neutral balsam. Images were captured using a digital slide scanner (NanoZoomer S60, HAMAMATSU).

**Immunofluorescent staining**

Immunofluorescent staining was performed using 10-30 µm sections. Section were deparaffinized, rehydrated and underwent antigen retrieval in similar ways as in IHC staining. Subsequently, sections were incubated in turn with primary at 4 overnight, and fluorescein-conjugated secondary antibodies mixed with DAPI (1:2000, 4083S, CST) for 1h at room temperature. Following washing using PBS, sections were mounted with buffer containing NaHCO3 (220.2 mM), Na2CO3 (28.3 mM) and 50% glycerol. Images were taken by a two-photon laser-scanning confocal microscope (AXR, Nikon).

**Morphological analysis of glia**

For microglia, 30-µm sections were scanned at 3-µm intervals in z-stack mode at 16-bit intensity resolution over 2048 × 2048 pixels, and an ortho image were acquired from projection based on the maximum intensity of a total of 10 images (AXR, Nikon). Reconstruction of 3D morphology of microglia was performed, and complexity of cell processes was measured by the principle of Sholl analysis^[2]^ via Imaris software (Version 9.8, Oxford Instrument). For astrocyte, 30-µm sections were stained and images were captured using a digital slide scanner (NanoZoomer S60, HAMAMATSU). Sholl analysis of cell processes were performed using Fiji software^[1]^. Equal number of glia cells were selected from different subregions of hippocampus for analysis, respectively.

**RT-qPCR**

Fresh frozen brain tissues were dissected and collected in sterile centrifuge tubes. Total RNA was purified from selected tissues using a TRIzol total RNA extraction reagent kit (Applygen, R1030) following the manufacturer’s instructions. For the detection of SARS-CoV-2 genome sequences, a SARS-COV-2 dual probes qRT-PCR kit (D8006S, Beyotime) was used to measure mRNA levels of SARS-CoV-2 nucleocapsid (N) and open reading frames 1ab (ORF1ab) coding region, using a LineGene 9600 Plus real time PCR detection system (LineGene 9600 Plus, Bioer Technology). SARS-CoV-2 pseudovirus was used as positive control. For the evaluation of inflammatory factors mRNA, a total of 5 μg RNA was used as template for reverse transcription. Then cDNA and GoTaq qPCR Master Mix (A6002, Promega) were used for RT-qPCR. The mRNA level of GAPDH was used as an internal control. Primers used here were summarized in supplementary Table S3.

**ddPCR**

ddPCR was performed using a SARS-CoV-2 nucleic acid detection kit (13444, TargetingOne) and Droplet Digital PCR system (TD-1, TargetingOne) following the manufacturers’ instructions. Briefly, 30-µL ddPCR mixture was prepared from 15 l RT-PCR mix with 15-µL solution containing RNA extracted from human brain samples or SARS-CoV-2 pseudovirus (positive control), followed by mixing with 180-µL oil buffer, and loading onto the droplet generation chip to produce droplets on a drop maker (TD-1, TargetingOne). The droplets were thermally cycled using the following protocol: 55°C for 15 minutes, then 95°C for 10 minutes, followed by 40 cycles of 94°C for 30 s, and 57°C for 1 min. The temperature ramp rate was set to 1.5°C/s on a thermal cycler (T100, Bio-Rad Laboratories). Finally, the droplets were detected on a chip reader and subjected to Poisson distribution analysis using the TargetingOne system (TD-1, TargetingOne). Primers used here were summarized in supplementary Table S3.

**Statistical analyses**

All data were analyzed and plotted using SPSS Statistics (IBM) or GraphPad Prism (GraphPad Software). One-way or repeated measures ANOVA followed by Tukey’s multiple comparisons tests were used, as indicated in each figure legend. *P* value less than 0.05 was considered significant, and a tendency in change was considered when 0.05 < *P* < 0.1 in some cases.

**References**

1. Schindelin J, Arganda-Carreras I, Frise E, Kaynig V, Longair M, Pietzsch T, Preibisch S, Rueden C, Saalfeld S, Schmid B, et al. Fiji: an open-source platform for biological-image analysis. Nat Methods. 2012;9:676-682
2. Sholl D. Dendritic organization in the neurons of the visual and motor cortices of the cat. J Anat. 1953;87:387-406

**Supplementary Table S1. Individual information of cases included in this study**

| Group | **#** | Gender | Age (y) | Regions | Comorbidity | Cause of death | PMI (h) | RDI (m) | Estimated ABC score |
| --- | --- | --- | --- | --- | --- | --- | --- | --- | --- |
| N | 1 | M | 58 | Hip, MEC | NA | Electrocution | 25 | ─ | A0, B1, C1 |
| N | 2 | M | 57 | Hip, MEC | NA | Methanol poisoning | 14 | ─ | A1, B0, C1 |
| N | 3 | F | 69 | Hip, MEC, AON, OT, Pir, LEC | Hypertension | Pulmonary embolism | 19 | ─ | A1, B0, C1 |
| N | 4 | F | 71 | Hip, MEC, AON, OT, Pir, LEC | Diabetes mellitus | Multiple organ dysfunction syndrome | 11 | ─ | A0, B2, C1 |
| N | 5 | M | 59 | Hip, MEC, OB, AON, OT, Pir, LEC | Coronary atherosclerosis | Acute myocardial infarction | 9 | ─ | A2, B1, C0 |
| N | 6 | F | 68 | Hip, MEC, OB, AON, OT, Pir, LEC | Coronary atherosclerosis, hyperlipidemia | Acute myocardial infarction | 15 | ─ | A1, B1, C1 |
| C | 1 | M | 74 | Hip, MEC | COVID19, hypertension | Heart failure | 28 | ─ | A2, B2, C1 |
| C | 2 | M | 71 | Hip, MEC | Covid19 | Heart failure | 21 | ─ | A1, B0, C0 |
| C | 3 | M | 69 | Hip, MEC, AON, OT, Pir, LEC | COVID19, Diabetes mellitus | Multiple organ dysfunction syndrome | 12 | ─ | A1, B2, C1 |
| C | 4 | F | 73 | Hip, MEC, AON, OT, Pir, LEC | COVID19, Hypertension, hyperlipidemia | Acute myocardial infarction | 11 | ─ | A1, B0, C0 |
| C | 5 | F | 68 | Hip, MEC, AON, OT, Pir, LEC | COVID19, Chronic pharyngitis, Hypertension, | Acute myocardial infarction | 35 | ─ | A0, B1, C0 |
| C | 6 | M | 73 | Hip, OB, AON, OT, Pir, LEC | COVID19, Chronic nephritis | Heart failure | 19 | ─ | A0, B2, C1 |
| C | 7 | M | 78 | Larynx, Lung | COVID19, Hyperlipidemia, Diabetes mellitus, | Acute myocardial infarction | 20 | ─ | A1, B1, C1 |
| R | 1 | F | 68 | Hip, MEC | Hypertension | Acute myocardial infarction | 32 | 4 | A1, B0, C0 |
| R | 2 | M | 48 | Hip, MEC | Pulmonary hypertension | Acute myocardial infarction | 28 | 6 | A0, B1, C1 |
| R | 3 | M | 59 | Hip, MEC | NA | Acute ethanol poisoning | 19 | 13 | A0, B2, C1 |
| R | 4 | M | 64 | Hip, MEC, AON, OT, Pir, LEC | Hypertension, Hyperlipidemia | Acute myocardial infarction | 26 | 7 | A1, B1, C1 |
| R | 5 | F | 49 | Hip, MEC, OB, AON, OT, Pir, LEC | Hepatitis C | Acute myocardial infarction | 14 | 10 | A0, B2, C1 |
| R | 6 | F | 64 | Hip, MEC, OB, AON, OT, Pir, LEC | NA | Hemorrhagic shock | 13 | 12 | A2, B1, C0 |
| R | 7 | M | 58 | Hip, MEC, OB, AON, OT, Pir, LEC | NA | Hemopneumothorax | 11 | 6 | A0, B0, C0 |

**Abbreviations:** Group: N, non-infected; C, COVID-19; R, recovery from COVID-19. Gender: F, female; M, male. Age (y), age (years). Comorbidity: NA, non-applicable. PMI (h), post-mortem interval (hours). RDI (m), recovery-death interval (months).

**Supplementary Table S2. Antibodies used in this study.**

| Antibody | Identifier and Source | Dilute and HIER |
| --- | --- | --- |
| rabbit anti-Tau-Phospho-Thr181 | 11107, SAB | IHC: 1:100; HIER: citrate buffer  WB: 1;1000 |
| rabbit anti-Tau-Phospho-Ser199 | 44-734G, Thermo Scientific | IHC: 1:100; HIER: citrate buffer  WB: 1;1000 |
| rabbit anti-Tau-Phospho-Thr205 | 11108, SAB | IHC: 1:100; HIER: citrate buffer  WB: 1;500 |
| rabbit anti-Tau-Phospho-Thr217 | 44-744, Thermo Scientific | IHC: 1:100; HIER: citrate buffer  WB: 1;500 |
| rabbit anti-Tau-Phospho-Thr396 | ab32057, Abcam | IHC: 1:100; HIER: citrate buffer  WB: 1;1000 |
| mouse anti-Tau-Phospho- Ser202-Thr205 (AT8) | MN1020, Thermo Scientific | WB: 1;250 |
| rabbit anti-Tau (Tau5) | ab80579, abcam | WB: 1;1000 |
| horseradish peroxidase conjugated mouse anti-β-actin antibody | AF0003, Biyuntian | WB: 1:2000 |
| rabbit anti-beta-Amyloid-chimeric-recombinant monoclonal antibody (6E10) | MA5-48043, Thermo Scientific | IHC: 1:100; HIER: citrate buffer |
| mouse anti-GFAP monoclonal antibody | 3070S, CST | IHC: 1:200; HIER: citrate buffer |
| goat anti-Iba1 antibody | ab5076, Abcam | IF: 1:200; HIER: citrate buffer |
| mouse anti-CD68 | 14-0688-82, Thermal Scientific | WB: 1:2000 |
| mouse anti-S100β | MA5-12969, Thermal Scientific | WB: 1:2000 |
| rabbit anti-SARS-CoV-2 nucleocapsid antibody | 40143-R001,Sino Biologicals | IHC: 1:3000; HIER: citrate buffer |
| rabbit anti-SARS-CoV-2 spike subunit 1 antibody | 40150-R007, Sino Biologicals | IHC: 1:200; HIER: citrate buffer |
| peroxidase-conjugated goat anti- rabbit secondary antibody | PV-6001, ZSGB-BIO | IHC: no dilution |
| peroxidase-conjugated goat anti-mouse secondary antibody | PV-6002, ZSGB-BIO | IHC: no dilution |
| Alexa-Fluor-488-conjugated donkey anti-goat secondary antibody | A-11055, Thermal Scientific | IF: 1:1000 |
| horseradish peroxidase conjugated goat anti-rabbit IgG antibody | ZB-5301, ZSGB-BIO | WB: 1:2000 |
| horseradish peroxidase-conjugated goat anti-mouse IgG antibody | ZB-2305, ZSGB-BIO | WB: 1:2000 |

**Supplementary Table S3. PCR primers used in this study.**

| Gene | Primer sequences | |
| --- | --- | --- |
|  | Forward | Reverse |
| GAPDH | ACT CAT GAC CAC AGT CCA TGC | GGC CAT CCA CAG TCT TCT GG |
| TNF-α | AGA GGG AGA GAA GCA ACT ACA | GGG TCA GTA TGT GAG AGG AAG A |
| IL-1β | CTC TCA CCT CTC CTA CTC ACT T | TCA GAA TGT GGG AGC GAA TG |
| IL-6 | GGA GAC TTG CCT GGT GAA A | CTG GCT TGT TCC TCA CTA CTC |
| IL-10 | GCT CCA AGA GAA AGG CAT CTA C | CCC TGA TGT CTC AGT TTC GTA TC |
| IL-18 | TCT TCA TTG ACC AAG GAA ATC GG | TCC GGG GTG CAT TAT CTC TAC |
| HMGB1 | TAT GGC AAA AGC GGA CAA GG | CTT CGC AAC ATC ACC AAT GGA |
| PAI1 | ACC GCA ACG TGG TTT TCT CA | TTG AAT CCC ATA GCT GCT TGA AT |
